# Histone deacetylase 6 plays an important role in TGF-β-induced murine Treg cell differentiation by regulating cell proliferation

**DOI:** 10.1038/s41598-022-27230-7

**Published:** 2022-12-29

**Authors:** Ji Hyeon Lee, Hyeong Su Kim, Sung Woong Jang, Gap Ryol Lee

**Affiliations:** Department of Life Science, Sogang University, 35 Baekbeom-Ro, Mapo-Gu, Seoul, 04107 Korea

**Keywords:** Immunology, Molecular biology

## Abstract

Regulatory T (Treg) cells maintain immune homeostasis by preventing abnormal or excessive immune responses. Histone deacetylase 6 (HDAC6) regulates expression of Foxp3, and thus, Treg cell differentiation; however, its role in Treg cell differentiation is unclear and somewhat controversial. Here, we investigated the role of HDAC6 in TGF-β-induced murine Treg cells. HDAC6 expression was higher in Treg cells than in other T helper cell subsets. Pharmacological inhibitors of HDAC6 selectively inhibited Treg cell differentiation and suppressive function. A specific HDAC6 inhibitor induced changes in global gene expression by Treg cells. Of these changes, genes related to cell division were prominently affected. In summary, HDAC6 plays an important role in TGF-β-induced murine Treg cell differentiation by regulating cell proliferation.

## Introduction

Regulatory T (Treg) cells belong to a subpopulation of T cells that maintain immune homeostasis^1–3^. In vivo, there are two types of Treg cells: thymus-derived Treg (tTreg) cells, which are derived from the thymus and migrate to peripheral tissues, and periphery-derived Treg (pTreg) cells, which are differentiated from naïve CD4 T cell in the periphery^4, 5^. Treg cells can also be generated in vitro by exposure to TGF-β: these cells are called iTreg cells^4, 5^. Treg cells play an essential role in peripheral tolerance and autoimmunity by regulating the activity of other immune cells^1, 6, 7^. Moreover, Treg cell-mediated suppression plays an important role in negative regulation of immune-mediated inflammation and autoimmune diseases^8^. By contrast, they also limit advantageous responses by repressing protective immunity against pathogens and by restricting antitumor immune responses^6, 9^. Therefore, abnormalities in Treg function are a fundamental cause of autoimmune and inflammatory disorders^10, 11^.

Functional Treg cells are characterized by specific expression of transcription factor forkhead and winged helix domain-containing protein 3 (Foxp3)^12, 13^. Foxp3 is a lineage-determining transcription factor that controls Treg development and function; thus, it is involved in immune homeostasis and is used as a major Treg marker^3, 14–16^. Foxp3 is an X chromosome-encoded transcription factor related to an X-linked fatal autoimmune disorder in humans called immunodysregulation polyendocrinopathy enteropathy X-linked syndrome (IPEX)^17–19^. Likewise, scurfy (sf) mice, an X-linked recessive mutant, show lymphoproliferative diseases and overexpression of numerous cytokines^12, 19^.

Epigenetic modifications, including DNA methylation and histone modifications, have a large impact on chromatin structure and gene expression in a heritable manner, but without changing genomic sequences^20, 21^. Studies show a general correlation between histone acetylation and gene activity, and that there are two classes of enzymes involved in determining the state of histone acetylation: histone acetyltransferases (HATs) and histone deacetylases (HDACs)^22–24^. HDACs are a class of evolutionarily conserved enzymes that remove acetyl groups from the lysine residues of histones and other proteins^25, 26^, allowing histones to wrap DNA more tightly. Tighter wrapping of DNA reduces the accessibility of transcription factors, resulting in transcriptional repression^25^. Humans possess 18 HDACs, which are classified into three main classes^27^, including HDAC6, which localizes to the cytoplasm and affects microtubule-dependent cell motility by functioning as a tubulin deacetylase^28^. It seems that HDAC6 mainly localizes in the cytoplasm, although it also interacts with some nuclear proteins including Foxp3 (reviewed in^29^). HDAC6 has been shown to interact with histones in vitro, but it was not confirmed in vivo^29^. Regulation of DNA methylation critically contributes to FOXP3 expression, lineage determination, and maintenance of Treg cells^30^. However, the link between histone deacetylation and FOXP3 expression remains unclear.

Repression of HDAC6 by pharmacological inhibitors or gene deletion increases Treg cell differentiation and function^31^. HDAC6 inhibitors suppress autoimmune diseases by damping down inflammation (reviewed in^32^). However, recent studies on the effect of HDAC6 inhibitors in tumor-infiltrating Treg cells are seemingly contradictory^33–35^. In these studies, HDAC6 inhibitors reduced Treg cell differentiation and function. Thus, the effect of HDAC6 inhibitors on Treg cells needs to be characterized more clearly.

Here, we examined the role of HDAC6 in TGF-β-induced murine Treg cell differentiation. A potent and highly selective HDAC6 inhibitor, Tubastatin A (TSA)^36, 37^, selectively downregulated the differentiation of Treg cells, but not that of Th1, Th2, and Th17 subsets. TSA reduced FOXP3 expression by Treg cells, leading to impairment of Treg identity and suppressive function. Moreover, RNA-sequencing (RNA-seq) analysis revealed that specific inhibition of HDAC6 affects the early differentiation stage and cell cycle phase of Treg cells. Overall, the results demonstrate that HDAC6 regulates lineage-specific differentiation of murine iTreg cells.

## Materials and methods

### Mice

Female C57BL/6 mice (aged 7–8 weeks) were purchased from Daehan Bio Link. All mice were housed under specific pathogen-free conditions and all animal experiments were approved by the Sogang University Institutional Animal Care and Use Committee (approval no. IACUCSGU2019_09). The reporting in this manuscript follows the ARRIVE guidelines.

### Differentiation of CD4 + T cell in vitro

Mice were used at age 7–10 weeks. Naïve CD4 + T cells were purified from mouse spleens using a MojoSort™ Mouse CD4 + Naïve T cell Isolation Kit (BioLegend). The isolated T cells were activated with plate-bound anti-CD3ε (145-2C11; 5 μg/ml) and soluble anti-CD28 (37.51; 2 μg/ml).

The following cytokines and antibodies were added to cell culture medium: for Th1 cell differentiation, mouse recombinant IL-2 (1 ng/ml), mouse recombinant IL-12 p70 (3.3 ng/ml), and 11B11 (anti-IL-4, 5 μg/ml); for Th2 differentiation, mouse recombinant IL-2 (1 ng/ml), mouse recombinant IL-4 (5 ng/ml), and XMG1.2 (anti-IFN-γ, 5 μg/ml); for Th17 cell differentiation, human recombinant TGF-β1 (1 ng/ml), mouse recombinant IL-6 (50 ng/ml), mouse recombinant TNFα (1 ng/ml), mouse recombinant IL-1β (10 ng/ml), XMG1.2 (5 μg/ml), and 11B11 (5 μg/ml); and for iTreg cell differentiation, mouse recombinant IL-2 (1 ng/ml), human recombinant TGF-β1 (5 ng/ml), XMG1.2 (10 μg/ml), and 11B11 (10 μg/ml). All cytokines used for differentiation were purchased from eBioscience.

TSA (a HDAC6 inhibitor) and ACY-738 (a HDAC6 inhibitor) were purchased from Selleckchem. HPOB (a HDAC6 inhibitor) and Nexturastat A (a HDAC6 inhibitor) were purchased from Cayman Chemical. Trichostatin A, a pan-HDAC inhibitor was purchased from Sigma-Aldrich. All inhibitors were solubilized in DMSO and added to culture media at a dilution of 1:1000.

### Splenic tTreg isolation and in vitro culture

Mice at age between 7 to 10 weeks were sacrificed and the spleens were isolated. After red blood cell lysis, cells were incubated with biotin anti-mouse CD8α (100,704, BioLegend), biotin anti-mouse I-A/I-E (107,604, BioLegend), biotin anti-mouse NK1.1 (108,704, BioLegend), biotin anti-mouse/human B220 (103,204, BioLegend), biotin anti-mouse CD49b (103,522, BioLegend), biotin anti-mouse CD19 (115,504, BioLegend), biotin anti-mouse/human CD11b (101,204, BioLegend), and biotin anti-mouse CD11c (117,304, BioLegend). Antibody-bound cells were then negatively selected by using MagnaBind™ Streptavidin (21,344, Thermo Fisher Scientific). Subsequently, Biotin anti-mouse CD25 (102,004, BioLegend) and MojoSort™ streptavidin nanobeads (480,016, BioLegend) were used for positive selection. The cells were purified by magnetic separation using LS Columns (130–042-401, Miltenyi Biotec) according to the manufacturer’s instructions.

The isolated cells were activated with plate-bound anti-CD3ε (145-2C11; 5 μg/ml) and soluble anti-CD28 (37.51; 2 μg/ml). For tTreg culture, mouse recombinant IL-2 (50 ng/ml), human recombinant TGF-β1 (5 ng/ml), XMG1.2 (10 μg/ml), and 11B11 (10 μg/ml) were added to the cell culture medium. All cytokines for differentiation were purchased from eBioscience.

### RNA isolation and quantitative real-time polymerase chain reaction (qRT-PCR)

Total RNA was extracted from cells using TRI-reagent (Molecular Research Center), according to the manufacturer’s instructions. Reverse transcription was carried out using TOPscript RT (Enzynomics). Next, a qRT-PCR assay was performed using TOPreal™ qPCR 2 × PreMIX TaqMan Probe or SYBR Green (Enzynomics) and a Roche LightCycler 96 instrument. The sequences of the primers used for qRT-PCR analysis are provided in Supplementary Table 1.

### Immunoblot analysis

After cell lysis using RIPA buffer (Sigma) containing a protease inhibitor cocktail (GenDEPOT), cell lysates were mixed with lane marker reducing sample buffer (Thermo Fisher Scientific) and boiled. The proteins were separated by SDS-PAGE and transferred to a PVDF membrane. The membrane was blocked for 1 h at room temperature (RT) with 5% skim milk prepared in TBS-T buffer. The membrane was then incubated overnight at 4 °C with a primary antibody diluted 1:1000 in 5% skim milk. After washing in TBS-T buffer, the membrane was incubated for 1 h at RT with an HRP-conjugated secondary antibody diluted 1:5000 in 5% skim milk. After washing again, signals were detected using West-Q Pico ECL solution or West-Q Femto clean ECL solution (GenDEPOT). An anti-HDAC6 antibody (D21B10, Cell Signaling Biotechnology) and an anti-β-actin antibody (C4, Santa Cruz Biotechnology) were used as the primary antibodies. HRP-conjugated anti-rabbit IgG and HRP-conjugated anti-mouse IgG were used as the secondary antibodies.

### Flow cytometry analysis

For intracellular cytokine staining, cells were re-stimulated for 4 h before harvest with phorbol myristate acetate (PMA) (50 ng/ml), ionomycin (1 μM) (both from Sigma-Aldrich), and Brefeldin A (BioLegend). Then, the cells were harvested, fixed, and permeabilized using an intracellular staining kit (eBioscience) prior to staining with PerCP/Cy5.5-conjugated anti-IL17A (506,919, BioLegend) and PerCP/Cy5.5-conjugated anti-Ki-67 (652,423, BioLegend) antibodies.

For transcription factor staining, cells were harvested directly, fixed, and permeabilized using a FOXP3 intracellular staining kit (BioLegend). Cells were then stained with a FITC-conjugated anti-Foxp3 antibody (11–5773-80, eBioscience) or an APC-conjugated anti-Foxp3 antibody (17–5773-82, eBioscience). Cells were also stained with APC-conjugated anti-CD152 (106,309, BioLegend), FITC-conjugated anti-GITR (120,205, BioLegend), PE-conjugated anti-ICOS (313,507, BioLegend), PE-conjugated anti-CD25 (102,008, BioLegend), and PerCP/Cy5.5-conjugated anti- programmed death-1 (PD-1; 135,207, BioLegend) antibodies. Stained cells were analyzed using an Accuri C6 Plus flow cytometer (BD Biosciences) or a FACSCalibur flow cytometer (BD Biosciences).

### In vitro suppression assay and proliferation assay

For the in vitro suppression assay, naïve T cells were treated with vehicle (DMSO) or a HDAC6 inhibitor (TSA), and then differentiated into iTreg cells for 3 days. Naïve T cells were stained with carboxy fluorescein succinimidyl ester (CFSE) (Sigma). Harvested iTreg and stained naïve T cells were cocultured in 96-well plates containing anti-CD3/CD28 beads (Invitrogen) (at several ratios). After 3 days, cells were harvested, and responder cells were selected and analyzed using a BD Accuri C6 Plus flow cytometer.

For the proliferation assay, naïve T cells were stained using CFSE (Sigma) and then polarized into each CD4 + T cell subset for 3 days. The stained cells were selected and analyzed using a BD Accuri C6 Plus flow cytometer.

### RNA-seq and gene set enrichment analysis (GSEA)

Total RNA was isolated using Trizol reagent (Invitrogen). RNA quality was assessed with an Agilent 2100 bioanalyzer using the RNA 6000 Nano Chip (Agilent Technologies, Amstelveen, the Netherlands), and RNA quantification was performed using an ND 2000 Spectrophotometer (Thermo Fisher, Waltham, MA, USA). For control and experimental RNAs, library construction was performed using the QuantSeq 3 mRNA Seq Library Prep Kit (Lexogen, Inc., Austria) according to the manufacturer’s instructions. High throughput sequencing was performed as single-end 75 sequencing using NextSeq 500 (Illumina, Inc., USA). RNA-seq data is available at GEO database (accession no. GSE 210,794). The RNA-seq analysis was performed with one set of biological samples and the number of differentially expressed genes was calculated based on more than twofold changes in gene expression level in TSA-treated and control Treg cells. Data analysis and graphic visualization were performed by ExDEGA (eBiogen Inc.). Gene classification was based on searches via Medline databases (https://www.ncbi.nlm.nih.gov/). GSEA from the Broad Institute (https://www.gsea-msigdb.org/gsea/index.jsp) was used to calculate enrichment of genes.

### Statistical analysis

Data are expressed as the mean ± standard deviation (SD). Differences between groups were determined by a two-way ANOVA or the Student’s *t* test, as appropriate. P values < 0.05 were considered statistically significant (*P < 0.05; **P < 0.01; ***P < 0.001; and ****P < 0.0001).

## Results

### iTreg cells express the highest amount of HDAC6 among CD4 T cell subsets

**Figure 1:**
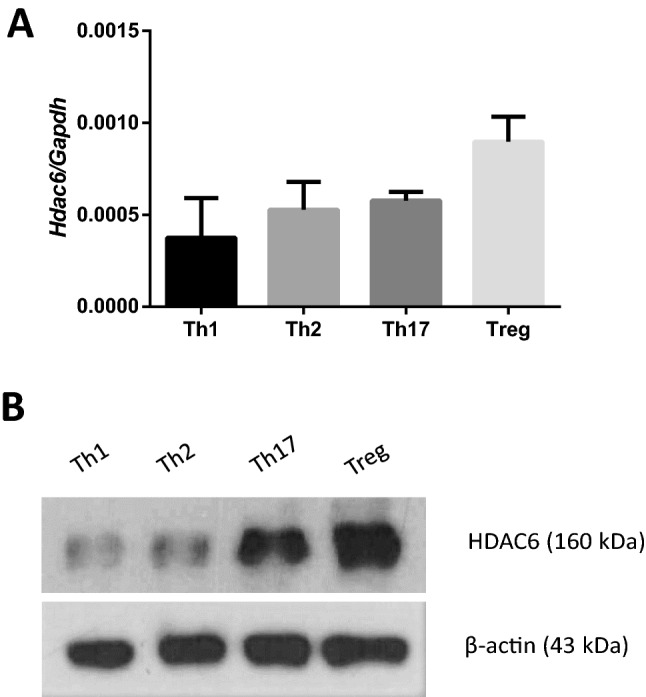
HDAC6 expression by different CD4 T cell subsets. (**A**, **B**) Naïve CD4 + T cells were isolated from mouse spleens and differentiated for 3 days into Th1, Th2, Th17, and Treg subsets. Relative expression of *Hdac6* mRNA was measured by qRT-PCR (**A**), and HDAC6 protein levels were measured by immunoblot analysis (**B**). qRT-PCR data are representative of three independent experiments. Error bars represent the SD.

To investigate the role of HDAC6 in each subtype of CD4 + T cells, we differentiated mouse naïve CD4 + T cells into Th1, Th2, Th17, or Treg cells and then measured the amount of HDAC6 mRNA (Fig. 1A) and protein (Fig. 1B, Suppl. Figure 1). At both the mRNA and protein levels, Treg cells showed the highest expression of HDAC6 among all CD4 + T cell subsets. This result suggests that HDAC6 may play an important role in Treg cell biology.

### A HDAC6 inhibitor regulates differentiation of Th17 and Treg cells in a dose-dependent manner

**Figure 2:**
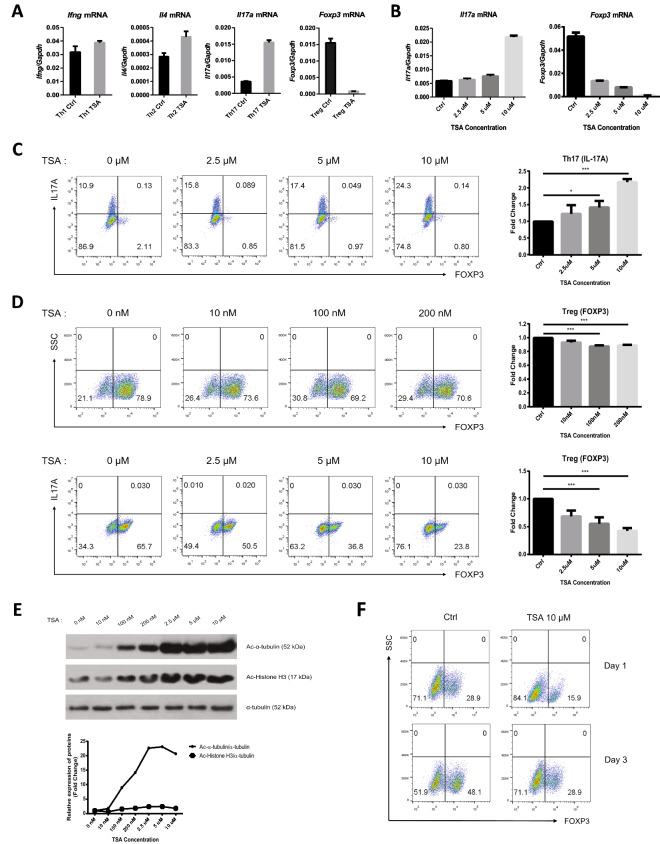
An HDAC6 inhibitor reduces Treg differentiation and maintenance. (**A**) Naïve CD4 + T cells were differentiated for 3 days into each CD4 + T cell subset in the presence of vehicle (control) or TSA (10 µM). Relative expression of *Ifng* mRNA by Th1 cells, *Il4* mRNA by Th2 cells, *Il17a* mRNA by Th17 cells, and *Foxp3* mRNA by Treg cells was measured by qRT-PCR. (**B**–**D**) Naïve CD4 + T cells were differentiated for 3 days into Th17 or Treg subsets in the presence of various concentrations of TSA. Relative expression of *Il17a* mRNA by Th17 cells, and *Foxp3* mRNA by Treg cells, was measured by qRT-PCR (B). The percentage of IL-17A + cells under Th17-polarizing conditions was analyzed by flow cytometry (**C**), and the percentage of FOXP3 + cells was analyzed under Treg-polarizing conditions (**D**). (**E**) Dose-dependent treatment of TSA increased the level of acetylated (Ac) α-tubulin. Protein levels of Ac-α-tubulin and Ac-Histone H3 were detected by immunoblot analysis (top) and relative expression of each protein was quantified using ImageJ software (below). (**F**) CD4 + CD25 + (tTreg) cells were isolated from mouse spleens and cultured for 1 or 3 days with vehicle (control) or TSA (10 µM). The percentage of FOXP3 + cells was analyzed by flow cytometry. qRT-PCR and dot plot data are representative of three independent experiments. Statistical analysis in C and D was performed using data pooled from three individual experiments. Error bars represent the SD, and P values were determined by the Student’s t test. *P < 0.05; **P < 0.01; ***P < 0.001; and ****P < 0.0001, n.s., not significant.

Each CD4 + T cell subset expresses lineage-specific transcription factors and cytokines that are important for effector function. For example, expression of T-bet and IFN-γ is associated with Th1 cells; that of GATA3 and IL4 with Th2 cells; that of RORrt and IL-17 with Th17 cells; and that of Foxp3 and IL-10 with Treg cells^38, 39^. To explore whether HDAC6 affects differentiation of CD4 + T cells, we differentiated naïve CD4 T cells into each subset in the presence of TSA, a selective HDAC6 inhibitor^36, 37^. Next, we performed qRT-PCR to measured expression of marker genes for each subset (Fig. 2A). Expression of *Ifng* mRNA in Th1 cells, and *Il4* mRNA in Th2 cells, increased slightly after TSA treatment. *Il17a* expression showed a significant increase in TSA-treated Th17 cells. By contrast, *Foxp3* mRNA levels in Treg cells fell significantly after TSA treatment (Fig. 2A). To further investigate reciprocal regulation of Th17 and Treg cells by TSA, we treated these cells with different concentrations (2.5–10 μM) of TSA and analyzed both mRNA (Fig. 2B) and protein (Fig. 2C and D) levels of each subset-specific marker gene. *Il17a* mRNA levels increased in Th17 cells, whereas *Foxp3* mRNA decreased in Treg cells, in a dose-dependent manner (Fig. 2B). Consistent with mRNA expression, expression of IL-17A protein increased in Th17 cells, and expression of FOXP3 protein decreased in Treg cells within a broad range of TSA concentration (10 nM ~ 10 μM), again in a dose-dependent manner (Fig. 2C and D). We examined the specificity of TSA on HDAC6 activity using α-tubulin and histone H3 as substrates. α-tubulin acetylation was increased dose-dependently by TSA treatment (0 to 10 μM range) up to 20 fold compared to untreated control. By contrast, histone H3 acetylation was only slightly increased by TSA treatment up to threefold. These results indicate that TAS specifically inhibited HDAC6 activity in the concentration used in this study (Fig. 2E, Suppl. Figure 2). When splenic Treg cells isolated from C57BL/6 mice were treated with TSA (10 μM)*,* Foxp3 expression decreased (Fig. 2F). Since splenic Treg cells are already differentiated cells, this result suggests that HDAC6 also affects maintenance of Treg cells by regulating Foxp3 expression. Collectively, the data suggest that TSA affects Th17 and Treg differentiation and maintenance in a dose-dependent manner.

### HDAC inhibitors repress iTreg cell differentiation

**Figure 3:**
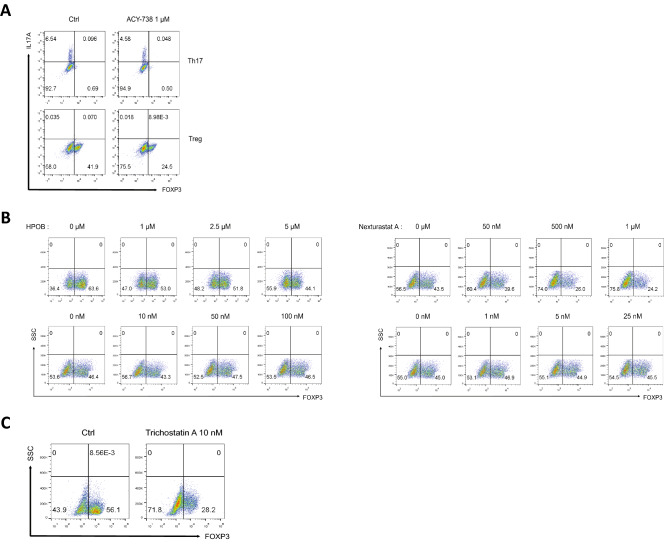
Various pharmacological inhibitors of HDAC6, or a pan-HDAC inhibitor, prevent iTreg differentiation. (**A**) Naïve CD4 + T cells were differentiated for 3 days into Th17 or Treg cells in the presence of vehicle control or ACY-738 (1 µM). The percentage of IL-17A + and FOXP3 + cells was analyzed by flow cytometry. (**B**) Naïve CD4 + T cells were differentiated for 3 days into Treg cells in the presence of various concentrations of HPOB (top) or Nexturastat A (bottom), and the percentage of FOXP3 + cells was analyzed by flow cytometry. (**C**) Naïve CD4 + T cells were differentiated for 3 days into Treg cells in the presence of vehicle control or Trichostatin A (10 nM). The percentage of FOXP3 + cells was analyzed by flow cytometry.

To confirm whether HDAC6, but no other members of the HDAC family, affects differentiation of Th17 and Treg cells, we tested independent HDAC6-specific inhibitors, ACY-738, HPOB, and Nexturastat A^40–43^. FACS analysis showed no change in IL-17A protein expression under Th17-skewing conditions, and a significant reduction in FOXP3 protein expression under Treg-skewing conditions, in the presence of ACY-738 (Fig. 3A). Moreover, HPOB and Nexturastat A decreased FOXP3 expression in Treg cells in a dose-dependent manner (Fig. 3B), which is consistent with previous results (Fig. 2). To further examine whether a pan-HDAC inhibitor has the same effects on Treg cell differentiation, we used the pan-HDAC inhibitor Trichostatin A^44^. When Treg cells were induced in the presence of Trichostatin A, Foxp3 protein levels fell by half (Fig. 3C). These data suggest that both HDAC6-specific and nonspecific HDAC inhibitors negatively regulate Foxp3 expression in iTreg cells.

### A HDAC6 inhibitor regulates the early stage of iTreg cell differentiation and impairs Treg cell identity

**Figure 4:**
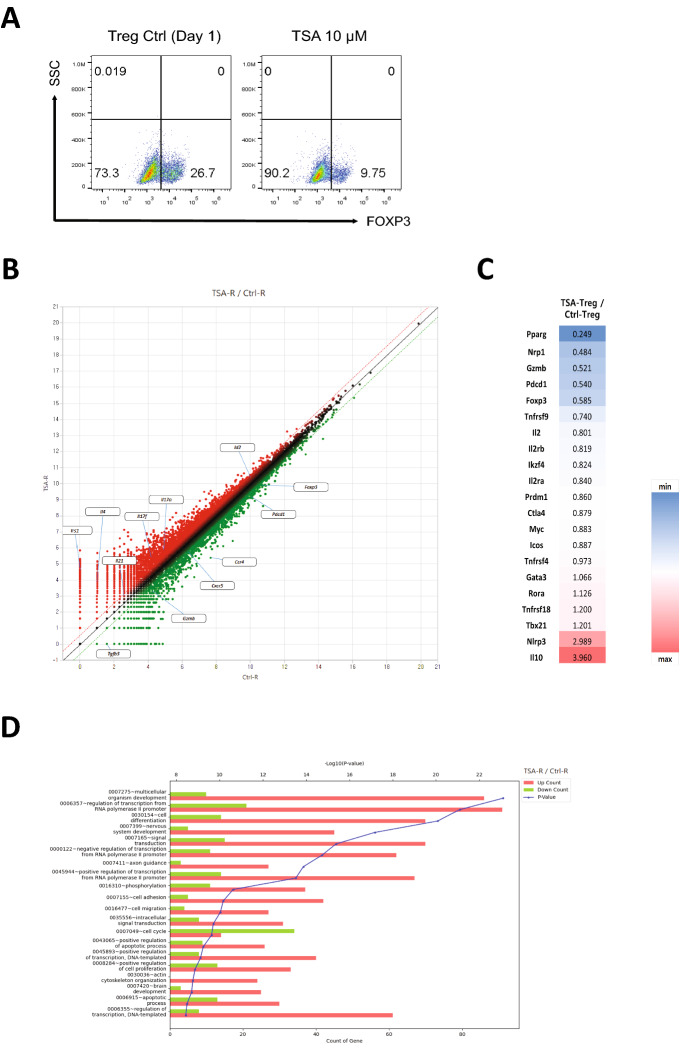
Global gene expression analysis by RNA-seq. (**A**) Naïve CD4 + T cells were differentiated for 1 day into Treg cells in the presence of vehicle (control) or TSA (10 μM). The percentage of FOXP3 + cells was analyzed by flow cytometry. (**B**) Scatter plot of RNA-seq data. RNA-seq analysis was conducted using total RNA isolated from control or TSA-treated Treg cells after 1 day of culture. Genes upregulated (-fold change > 2.0) are shown in red; genes downregulated (-fold change > 2.0) are shown in green. (**C**) Heat map of -fold changes shows expression of Th- and Treg-associated genes. Red and blue represent high and low levels of expression of the indicated genes, respectively. (**D**) GO analysis of differentially expressed genes in control or TSA-treated Treg cells.

To explore whether TSA has effects at the early stage of iTreg differentiation, we treated naïve CD4 + T cells with DMSO (vehicle control) or 10 μM TSA and then cultured them for 1 day under Treg-inducing conditions. The percentage of FOXP3 + cells decreased by more than half upon TSA treatment (Fig. 4A). Next, we performed RNA-seq to gain insight into the different transcription profiles of control and TSA-treated Treg cells. Based on more than twofold changes in gene expression level in TSA-treated and control Treg cells, we identified 2,722 differentially expressed genes in TSA-treated cells (Fig. 4B). Among 907 downregulated transcripts were Treg signature genes such as *Foxp3, Pdcd1, Ccr4,* and *Cxcr5.* By contrast, expression of inflammatory cytokines, including *Il17a, Il17f., Il4,* and *Il21*, by effector CD4 + T cells was upregulated (Fig. 4B). We then selected genes associated with conventional T (Tconv) cells or Treg cells, and found that TSA-treated Treg cells seemed to fail to differentiate into Treg cells; indeed, these cells showed higher expression of lineage-determining transcription factors associated with Tconv cells, and lower expression of Treg marker genes (Fig. 4C). PPAR-γ negatively regulates Th17 differentiation^45^ and acts as an essential molecule that manages visceral adipose tissue Treg cell accumulation, phenotype, and function^46^. Moreover, NLRP3, a crucial factor for inflammasome formation^47^, plays a role as a negative regulator of Treg differentiation^48^. These gene expression profiles suggest that TSA-treated cells fail to differentiate to Treg cells. We then used DAVID Gene Ontology (GO) analysis (http://david.ncifcrf.gov) to investigate gene categories that are altered in TSA-treated Treg cells. We found alterations in genes associated with cell differentiation, signal transduction, regulation transcription, and the cell cycle (Fig. 4D). A previous study shows that HDAC6 may be recruited to chromatin through physical interaction with phosphorylated RNA polymerase II^49^. Our data also suggest that many genes related to regulation of transcription via the RNA polymerase II promoter were altered significantly (Fig. 4D). Taken together, these results suggest that HDAC6 regulates many genes involved in iTreg cell differentiation.

### HDAC6 inhibitors hinder the immunosuppressive function of iTreg cells

**Figure 5:**
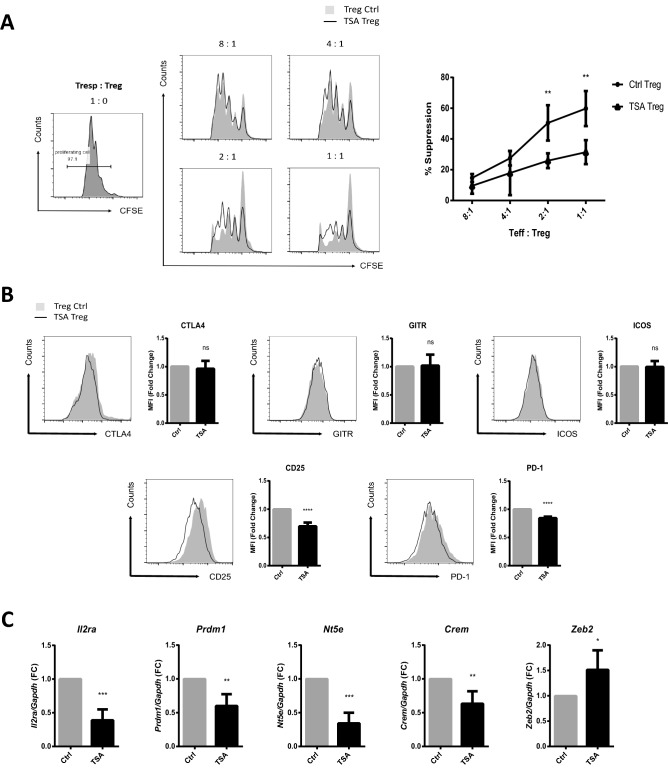
An HDAC6 inhibitor reduces iTreg cell proliferation. (**A**) Naïve CD4 + T cells were stained with CFSE and cocultured with the indicated ratios of Treg cells. The in vitro immunosuppressive activity of Treg cells was quantified by analyzing proliferation of naïve CD4 + T cells. Histogram data are representative of three independent experiments, and statistical analysis was performed using data pooled from three independent experiments. (**B**) Naïve CD4 + T cells were differentiated for 3 days into Treg cells in the presence of vehicle (control) or TSA (10 μM). Expression of CTLA4, GITR, ICOS, CD25, and PD-1 protein was analyzed by flow cytometry. The MFI for each experiment was measured and data from four independent experiments were pooled. (**C**) Cells were cultured as described in B. Expression of *Il2ra, Prdm1, Nt5e, Crem,* and *Zeb2* mRNA was measured by qRT-PCR. qRT-PCR data from four independent experiments were pooled. Error bars represent the SD, and P values were determined by a two-way ANOVA (A) and the Student’s t test (**B**, **C**). *P < 0.05; **P < 0.01; ***P < 0.001; and ****P < 0.0001, n.s., not significant.

To examine whether HDAC6 affects the functional properties of iTreg cells, we conducted an in vitro suppression assay. Naïve CD4 T cells were differentiated into Treg cells in the presence or absence of 10 μM TSA. CFSE-labeled naïve CD4 T cells were used as responder T (Tresp) cells. Tresp cells were mixed with Treg cells at various ratios and then cocultured in the presence of αCD3/αCD28 beads for 3 days. Finally, proliferation of Tresp cells was measured by flow cytometry. CFSE-stained Tresp cells proliferated to a greater extent when cocultured with TSA-treated Treg cells than when cocultured with control Treg cells (Fig. 5A). To explore the molecular mechanism underlying the reduced suppressive activity of Treg cells in the presence of TSA, we examined their cell surface phenotype. Flow cytometry analysis revealed that Treg cells with or without TSA treatment expressed similar levels of CTLA4, GITR, and ICOS on the surface (Fig. 5B). However, expression of CD25, a key marker of suppressive CD4 + T cells^1^, fell significantly. Expression of PD-1, which controls Treg cell development and function^50^, was also reduced by TSA. Furthermore, as expression of Foxp3 decreased, expression of Foxp3 target genes also changed. Foxp3 can to bind to DNA and regulate transcription of other factors and therefore plays a central role in establishing the Treg lineage^13^, both directly and indirectly. *Il2ra, Prdm1, Nt5e,* and *Crem* in Treg cells are upregulated by Foxp3^51, 52^, whereas *Zeb2* is repressed^53^. This is consistent with our finding that TSA-treated Treg cells showed reduced expression of *Il2ra, Prdm1, Nt5e,* and *Crem,* and elevated expression of *Zeb2* (Fig. 5C). To examine whether RORγt was affected by TSA treatment in Treg cells, we measured expression of *Rorc* mRNA level in Treg cells and Th17 cells. TSA-treated Treg cells did not increase expression of *Rorc* mRNA, although TSA-treated Th17 cells increased *Rorc* mRNA expression. These results suggest that reduction of Foxp3 expression in TSA-treated Treg cells was not due to increased *Rorc* expression (Suppl. Figure 3). Collectively, these results suggest that TSA-treated iTreg cells are less suppressive than control Treg cells, and that they undergo changes in global gene expression that reflect failure of Treg cell differentiation.

### HDAC6 inhibitors suppress cell cycle progression in iTreg cells

**Figure 6:**
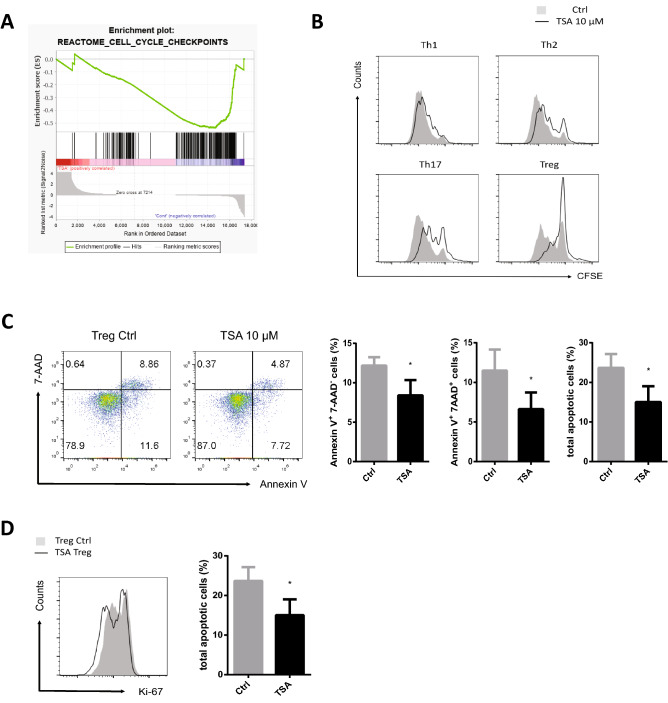
Reduced proliferation in the presence of the HDAC6 inhibitor is due to diminished proliferation rather than to increased apoptosis. (**A**) GSEA plot of genes related to cell cycle checkpoints. (**B**) Naïve CD4 + T cells were stained with CFSE and differentiated for 3 days into each CD4 + T cell subset in the presence of vehicle (control) or TSA (10 μM). The proliferation of each CD4 + T cell subset was quantified by measuring CFSE fluorescence. (**C**) Naïve CD4 + T cells were differentiated for 3 days into Treg cells in the presence of vehicle (control) or TSA (10 μM). Apoptosis of Treg cells was measured by flow cytometry after Annexin V/7-AAD staining. (**D**) Cells were cultured as described in C. Ki-67 protein expression was analyzed by flow cytometry. Data shown in the dot plot and histogram are representative of four independent experiments. Statistical analysis in C and D was performed using data pooled from four independent experiments. Error bars represent the SD, and P values were determined by the Student’s t test. *P < 0.05; **P < 0.01; *** P < 0.001; and ****P < 0.0001, n.s., not significant.

To further explore alterations of Treg function, we performed GSEA using RNA-seq data (GSE210794). GSEA revealed that genes related to the cell cycle checkpoint were positively enriched in TSA-treated Treg cells (Fig. 6A). This result is consistent with the GO analysis (Fig. 4D). To examine whether Treg cells are more susceptible to TSA than other subsets, proliferation of each CD4 + T cell subset was monitored in a CFSE dilution assay in the presence or absence of TSA. Although division of all CD4 + T cell subsets was suppressed by TSA, Treg cells showed the greatest reduction in proliferation (Fig. 6B). To examine whether this is due to apoptosis, we measured apoptosis by Annexin V and 7-AAD staining. The numbers of early apoptotic cells (Annexin V + /7-AAD-), late apoptotic cells (Annexin V + /7-AAD +), and total apoptotic cells in the TSA-treated group were all lower than those in the control group (Fig. 6C). In addition, we measured expression of Ki-67, which is widely used as a marker of cell proliferation. The mean fluorescence intensity (MFI) value for Ki-67 was significantly decreased in the TSA-treated Treg group (Fig. 6D). Thus, the reduced percentage of divided Treg cells was not due to apoptosis, but to inhibition of cellular proliferation.

## Discussion

Treg cells are a distinct subset of CD4 + T cells that prevents abnormal or excessive immune responses and development of autoimmune disorders. However, because they also suppress other effector T cells, depleting Tregs can be clinically beneficial in some cancer models. Thus, proper regulation of Treg cell differentiation and function is a promising therapeutic approach to diverse diseases. Because Foxp3 is a key transcription factor that is essential for differentiation and inhibitory function of Treg cells, it is important to understand the molecular mechanisms that control Foxp3 induction and maintenance. Although we know how epigenetic modifications influence gene regulation in general, it is not clear how HDACs regulate Foxp3.

Here, we show that HDAC6 is an important regulator of murine iTreg cell differentiation and function. Among all CD4 + T cell subsets examined, HDAC6 mRNA and protein levels were highest in Treg cells. We used HDAC6-selective inhibitors to examine the effect of HDAC6 on Treg cells. Whereas expression of genetic markers specific for conventional CD4 + T cells increased upon exposure to TSA, that of *Foxp3* in Treg cells fell significantly. Moreover, when treated with different concentrations of TSA, expression of IL-17A protein in Th17 cells increased, but that of Foxp3 in Treg cells decreased, in a dose-dependent manner. A previous study shows that deficiency of HDAC6 promotes IL-17A production by γδ T cells^54^. These data suggest that HDAC6 may affect reciprocal regulation of Th17 and Treg cells. This reduction in Foxp3 expression was also noted after exposure to other HDAC6 inhibitors (i.e., ACY-738, HPOB, and Nexturastat A) and a pan-HDAC inhibitor (Trichostatin A). These results show that HDAC6 is required for Foxp3 induction.

Next, we performed RNA-seq analysis to identify changes in global gene expression after HDAC6 inhibition. We found that TSA-treated iTreg cells lose the characteristics of Treg cells. Changes in transcript levels led to functional changes in Treg cells. Loss of Foxp3 expression, along with that of surface markers such as CD25 and PD-1, attenuated the suppressive capacity of TSA-treated Treg cells in vitro*.* Increased expression of PD-1 contributes to the suppressive function of Treg cells^55^. Furthermore, GSEA analysis suggests that, compared with control Treg cells, genes highly expressed by TSA-treated Treg cells are enriched in the category “cell cycle checkpoints”. Inhibiting HDACs alters many biological processes that affect gene expression, cell proliferation, differentiation, and cell survival^56^. Our results also suggest that proliferation and expression of related makers in Treg cells falls markedly after treatment with TSA, while the rate of apoptosis falls slightly.

Our data show that selective HDAC6 inhibitors inhibit Treg cell differentiation in vitro*.* This result is in stark contrast with that presented in a previous report showing that gene deletion or a pharmacological inhibitor of HDAC6 increases expression of Foxp3, and increases their suppressive capability^31^. By contrast, recent studies show that Treg frequency falls and tumor growth is inhibited after treatment with selective HDAC6 inhibitors^33–35^, which supports our results. In accordance with these findings, selective inhibition of HDAC6 shows potential as an effective cure in various tumor models^57, 58^. It is not clear why different studies report different effects of HDAC6 inhibition. Differences in the method of T cell activation may be one possible reason. The use of antigen-presenting cells, in particular, in the inhibitor-treated culture medium can have indirect effect of the inhibitor exerted on antigen-presenting cells. Another possible reason is different concentrations of the pharmacological inhibitors used in different studies because high concentrations may affect other HDACs in addition to the specific target. Further studies are needed to obtain more consistent results.

In summary, we show here that pharmacological inhibition of HDAC6 impairs murine iTreg cell function by downregulating Foxp3 expression. Our findings also suggest the possibility that HDAC6 might be a potential therapeutic target in inflammatory diseases. Given that TSA inhibits the suppressive ability of Treg cells, treatment with TSA may be an effective therapeutic strategy for curing immune-related diseases and tumors by amplifying the effects of effector T cells and other antitumor immune responses.

## Supplementary Information

Supplementary Information.
