# Supplementary material for: Histone deacetylase 6 plays an important role in TGF-β-induced murine Treg cell differentiation by regulating cell proliferation
Source: Sci Rep. 2022 Dec 29;12:22550. doi: 10.1038/s41598-022-27230-7 (PMC9800578; doi:10.1038/s41598-022-27230-7)
Supplement: Supplementary file 1 — Supplementary Information. [file 41598_2022_27230_MOESM1_ESM.docx]

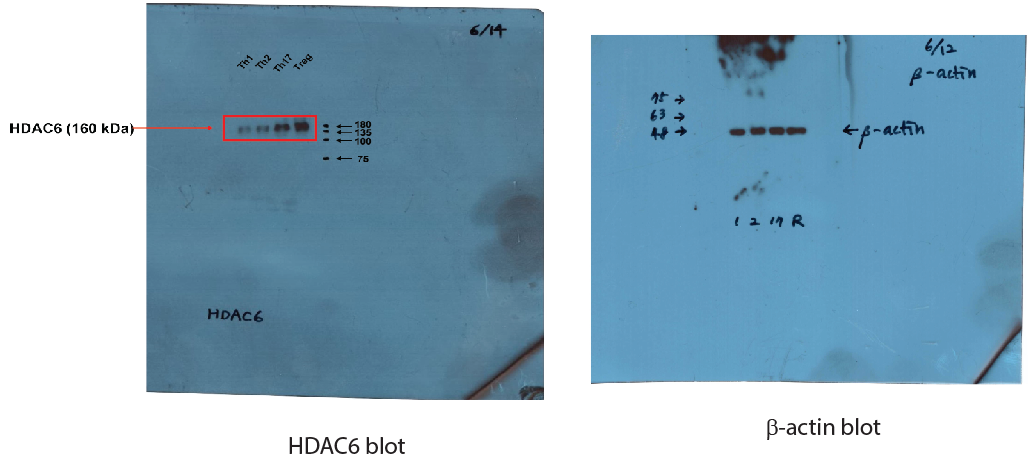


**Supplementary Figure 1. Whole blots of Fig 1B.**

Left panel: HDAC6 blot, right panel: β-actin blot.


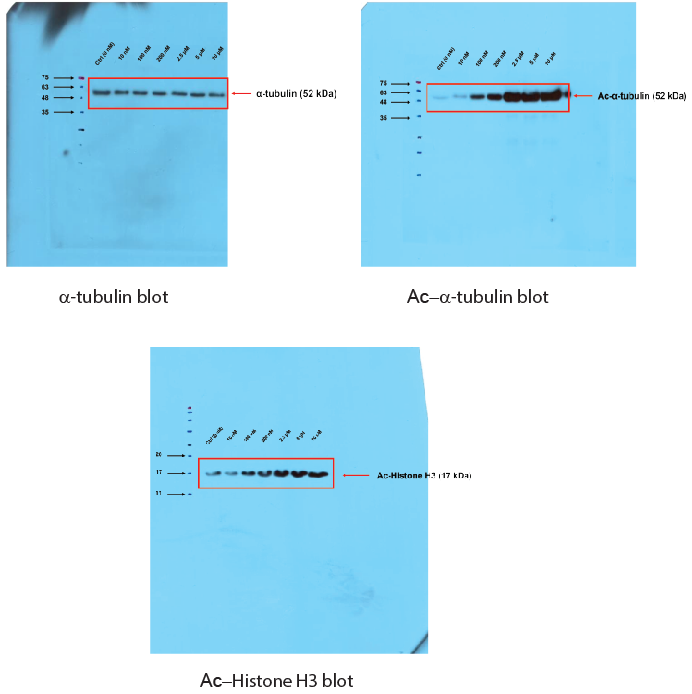


**Supplementary Figure 2. Whole blots of Fig 2E.**

Upper left panel: α-tubulin blot, upper right panel: Ac-α-tubulin blot, lower panel: Ac-Histone H3 blot


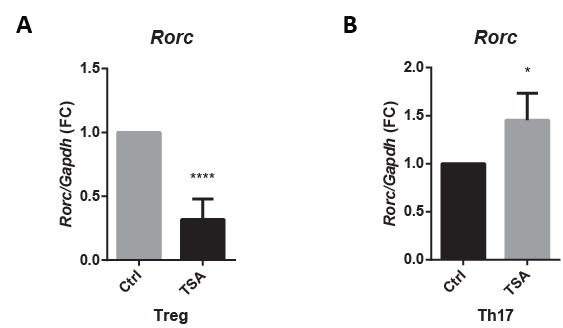


**Supplementary Figure 3. Rorc expression in TSA-treated Treg cells (A) and Th17 cells (B).**

Naïve CD4+ T cells were differentiated into Treg or Th17 subset with vehicle (control) or TSA (10 μM) for 3 days. Relative expression of *Rorc* mRNA was measured by qRT-PCR. Statistical analysis in A was performed on pooled data from five independent experiments and in B on pooled data from three independent experiments. Error bars represent the SD, and P values were determined by the Student’s t test. *, P < 0.05; **, P < 0.01; ***, P < 0.001; ****, P < 0.0001, n.s., not significant.

**Supplementary Table 1. Primers list used for qRT-PCR**

| Primer name | Sequences (5´→3´) |
| --- | --- |
| *Ifng* Forward primer | GGATGCATTCATGAGTATTGC |

| *Ifng* Reverse primer | CCTTTTCCGCTTCCTGAGG |
| --- | --- |
| *Ifng* probe | TTTGAGGTCAACAACCCACAGGTCCA |
| *Il4* Forward primer | AGATCATCGGCATTTTGAACG |
| *Il4* Reverse primer | TTTGGCACATCCATCTCC |
| *Il4* probe | TCACAGGAGAAGGGACGCCATGC |
| *Il17a* Forward primer | CTCCAGAAGGCCCTCAGACTAC |
| *Il17a* Reverse primer | AGCTTTCCCTCCGCATTGACACAG |
| *Il17a* probe | TCTGGGAAGCTCAGTGCCGCCACCAGC |
| *Foxp3* Forward primer | CCCAGGAAAGACAGCAACCTT |
| *Foxp3* Reverse primer | TTCTCACAACCAGGCCACTTG |
| *Foxp3* probe | ATCCTACCCACTGCTGGCAAATGGAGTC |
| *Gapdh* Forward primer | CAATGTGTCCGTCGTGGATCT |
| *Gapdh* Reverse primer | GTCCTCAGTGTAGCCCAAGATG |
| *Gapdh* probe | CGTGCCGCCTGGAGAAACCTGCC |
| *Hdac6* Forward primer | TCCACCGGCCAAGATTCTTC |
| *Hdac6* Reverse primer | CAGCACACTTCTTTCCACCAC |
| *Il2ra* Forward primer | AACCATAGTACCCAGTTGTCGG |
| *Il2ra* Reverse primer | TCCTAAGCAACGCATATAGACCA |
| *Prdm1* Forward primer | AAGACGTTCGGTCAGCTCTCCA |
| *Prdm1* Reverse primer | CTGGCACTCATGTGGCTTCTCT |
| *Nt5e* Forward primer | GGACATTTGACCTCGTCCAAT |
| *Nt5e* Reverse primer | GGGCACTCGACACTTGGTG |
| *Crem* Forward primer | CAGACTAGCACGGGGCAATAC |
| *Crem* Reverse primer | AGCCACACGATTTTCAAGACAT |
| *Zeb2* Forward primer | ATTGCACATCAGACTTTGAGGAA |
| *Zeb2* Reverse primer | ATAATGGCCGTGTCGCTTCG |
| *Rorc* Forward primer | ATGAGAACACAAATTGAAGTGATCC |
| *Rorc* Reverse primer | AGTAGGCCACATTACACTGCT |
